# Novel Approach, Using End-of-Life Issues, for Identifying Items for Public Health Surveillance

**Published:** 2009-03-15

**Authors:** Jaya K. Rao, Lindsay A. Abraham, Lynda A. Anderson

**Affiliations:** University of North Carolina Eshelman School of Pharmacy. At the time that this work was performed, Dr Rao was affiliated with the Centers for Disease Control and Prevention and Emory University School of Medicine, Atlanta Georgia.; Centers for Disease Control and Prevention, Atlanta, Georgia; Centers for Disease Control and Prevention, and Emory University Rollins School of Public Health, Atlanta, Georgia

## Abstract

Using end-of-life (EOL) issues to provide context, we introduce a novel approach to identify potential items for public health surveillance. Our method involved an environmental scan of existing EOL surveys and included the following steps: 1) consulting experts for advice on critical EOL topics, 2) identifying a broad sample of EOL surveys, and 3) using an abstraction tool to characterize surveys and survey items. We identified 36 EOL surveys; of these, 10 were state-based surveys. Of the 1,495 EOL items (range, 4 to 126 items per survey), 333 items could be classified in 1 of 11 topic areas of interest. Information on the surveys and these 333 items was entered into a database. As a result of this process, we identified topics for which many EOL items already exist and topics for which items should be developed. We describe the value of this approach and potential next steps for our project.

## Introduction

Life expectancy in the United States increased by 30 years during the last century (1,2). Most people alive today will die at an older age than in previous years, most likely after a period of chronic illness and physical decline (3,4). At the same time, studies document serious deficiencies in the care provided to dying people, including undertreatment of pain and communication difficulties between patients, family members, and health care providers regarding end-of-life (EOL) goals (5-7). As a result, EOL issues have gained recognition as a societal (8) and public health (9) concern.

The Institute of Medicine (10) and the National Institutes of Health (11) have emphasized the need for data that can be used to improve the experiences of dying people and their families. In making these recommendations, both organizations focused on data that could be used to improve the experiences of dying people within health care systems. However, they recommended that federal agencies "make incremental changes to [existing] surveys to improve the usefulness of currently collected data in describing aspects of quality of life and quality of care at the end of life" (10). To date, EOL items have not been included in surveillance systems that address issues that affect quality of life. In 2003, 1 of the top 5 recommendations made by stakeholders from diverse fields concerning the role of state health departments in addressing EOL issues was a recommendation to "collect, analyze, and share data about EOL through state surveys, such as the Behavioral Risk Factor Surveillance System" (BRFSS) (12).

Before creating EOL items to address this recommendation, we recognized the need to determine whether items that are appropriate for ongoing public health surveys or surveillance systems already existed and to identify gaps that require the development of new items. To meet these goals, we developed a systematic approach that involved an environmental scan or a search for existing EOL instruments or surveys (hereafter referred to as surveys) and abstracting items within key topic areas.

## Methods

As a first step (Figure), we convened a 1-day meeting in February 2006 with 6 people who had expertise in palliative care, EOL survey research, or public health, or some combination of the 3. At the beginning of the meeting, we gave a presentation on public health surveillance methods and how data collected using these methods are used to inform public health activities. Then, we asked the experts to identify and prioritize key EOL topic areas that are appropriate to incorporate into ongoing public health surveys or surveillance systems. The panel members identified 8 critical topics: 1) awareness of EOL options, 2) communications with family members and health care providers about EOL preferences, 3) communications with family members and health care providers about advance directives, 4) general concerns and fears about dying, 5) desires regarding EOL care, 6) location of death of a loved one, 7) unmet needs at the end of the person's life, and 8) pain at the end of the person's life.

**Figure.**
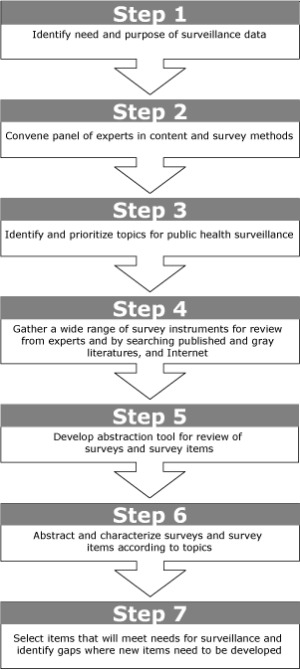
Step-by-step method for identifying potential survey items for public health surveillance from existing surveys.

**Table:** 

The figure depicts a series of 7 boxes, which arranged linearly so that the read from top to bottom. Each box is connected with the box beneath it with a downward-pointing arrow.
The first box reads, “Identify need and purpose of surveillance data,” and it is connected with box 2, which is positioned directly below it. The second box reads, “Convene panel of experts in content and survey methods,” and it is connected with box 3, which is positioned directly below it. The third box reads, “Identify and prioritize topics for public health surveillance,” and it is connected with box 4, which is positioned directly below it. The fourth box reads, “Gather a wide range of survey instruments for review from experts and by searching published and gray literatures, and Internet,” and it is connected with box 5, which is positioned directly below it. The fifth box reads, “Develop abstraction tool for review of surveys and survey items,” and it is connected with box 6, which is positioned directly below it. The sixth box reads, “Abstract and characterize surveys and survey items according to topics,” and it is connected with box 7, which is positioned directly below it. The seventh and final box reads, “Select items that will meet needs for surveillance and identify gaps where new items need to be developed.”

Because one of our goals was to identify existing EOL items, we needed to collect and systematically review a broad sample of EOL surveys for items related to these key topic areas. We used an iterative approach for this review to identify surveys that were conducted after 1990. We searched the Internet and published and "gray" literature (articles, technical reports, newsletters, or other documents produced by government agencies, academic institutions, and other groups not indexed or distributed by commercial publishers), and we consulted expert panel members. In addition to providing us with their own surveys, the experts facilitated contacts with other researchers who provided us with additional instruments. Given our interest in identifying items suitable for population-based surveillance, we excluded surveys that focused solely on the processes of care (eg, surveys of health care institutions) or health care professionals or surveys that were not performed in English.

Next, we developed an abstraction tool. Our tool captured information on 2 levels: 1) characteristics of the survey and 2) characteristics of individual items. For each survey, we collected information on the sampling frame (national, state, community, hospital, hospice, or nursing home), type of sample (ie, general public, patient, or family member), and mode of administration (telephone, in-person, written, or multiple modes). Next, we abstracted and classified each survey item according to 1) perspective (retrospective or prospective), 2) response type, and 3) topic. In terms of perspective, we classified items as retrospective if they asked respondents to provide information on the experiences of a family member or significant other who died within a specified time frame (a mortality follow-back approach) (13). We classified items as prospective if they assessed the respondents’ personal awareness, attitudes, and behaviors related to EOL issues. We used the following categories to characterize item response types: Likert-type, multiple-choice, open-ended, ranking, rating, or yes/no response. Our topic categories included the 8 areas identified by the expert panel members and an additional 3 areas (completion of advance directive, concerns about being a burden to others, and health care provider communications with the dying person or family member). All 11 topics are relevant to our efforts to develop and track public health EOL programs.

As part of the abstraction process, 2 authors classified survey items into 4 overarching categories, which contained the 11 topics. The first category, labeled "knowledge," consisted of 1 topic, awareness of EOL options. The second category, labeled "attitude," included 3 topics: concerns about being a burden to others, general concerns and fears about dying, and desires regarding EOL care. The third category, labeled "behavior," included 3 topics: discussion about EOL preferences, communication about advance directives, and completion of advance directive. The final category, labeled "situation," included 4 topics: location of death of a loved one, how well this person's needs were met, whether the person experienced pain at the end of his or her life, and health care provider communications with the dying person or family member. All items were classified into mutually exclusive categories and topics (items were classified into 1 of the 4 overarching categories and 1 of 11 topic areas). In some instances, we found that different surveys included the same item. We abstracted these items only once and recorded the surveys in which they appeared. Any differences between the raters in the classification of surveys or survey items were discussed and resolved before data were entered into the study database.

## Results

We identified 36 surveys for our environmental scan (Table 1). Because our search strategy included the published and gray literature, the list of surveys included in our review is available from the authors. Of the surveys, most (n = 31, 86%) were performed in the United States; the remaining 5 were conducted in Canada (n = 4) or Australia (n = 1). In terms of sampling frame, 18 surveys were performed at the national or state level, and the remainder were performed in communities, hospitals, hospices, or nursing homes. Among the national surveys, 3 were public opinion polls, 3 were specific waves of population-based surveys (eg, Longitudinal Survey on Aging, Health and Retirement Study) in which some EOL items were included, and 2 were national mortality follow-back surveys. We found 3 state-added EOL modules for the BRFSS and 7 other surveys that were performed in specific states.

The types of respondents varied considerably. In 20 surveys, the respondents were members of the general public, 11 surveys focused on family members, and 5 surveys were administered to various patient groups. Various methods were used to administer these surveys: in-person administration was most common (n = 14), followed by telephone, written, and telephone and written modes of administration (Table 1).

The 36 surveys contained a total of 1,495 EOL items, covering a wide range of issues (range, 4 to 126 items per survey). Some items were duplicated across surveys. For example, 3 pairs of surveys contained identical items (n = 55), and an additional survey included items from 5 other surveys (n = 30). Of the 1,410 unique EOL items, 333 could be classified in 1 of the 11 topic areas; the remaining items (n = 1,077) did not relate to our topic areas and focused mostly on clinical symptoms experienced by the dying person or specific details about the quality of EOL care received. Overall, we found slightly more retrospective items than prospective items (174 vs 159).

Of the 333 EOL items, 260 were classified in the situation (n = 136) or attitude (n = 124) categories; fewer items were classified in the knowledge and behavior categories (Table 2). The knowledge and situation categories contained items with only 1 perspective (either retrospective or prospective), whereas the attitude and behavior categories included both retrospective and prospective items. The knowledge items elicited information on respondents' awareness of various EOL options, such as hospice and palliative care, the Medicare hospice benefit, and advance care planning (data not shown). Of the 124 attitude items, 75 fit within the desires topic and examined different expectations the respondent might have for EOL care (eg, where he or she would like to die, types of care desired, use of life-sustaining treatments).

Nearly all of the items in the behavior category were classified in 2 topic areas: completion of an advance directive (n = 36 items) and discussion of EOL preferences and options with others (n = 17 items). Few items (n = 4) focused on specific communications related to advance directives. Finally, of the 136 items in the situation category, 60 fit in the needs topic area, and the remaining items were evenly distributed between the location of death (n = 24 items), pain at the end of the patient's life (n = 25 items), and provider communication (n = 27 items) topics. The needs items examined various issues, including the degree to which the dying person's symptoms were controlled and whether spiritual and psychological support was available to the dying person and family members.

## Discussion

Once the public health community recognizes the need for surveillance of a particular health issue, developing suitable items for population-based surveys takes time and resources. We conducted an environmental scan to identify and characterize existing survey items that may be appropriate for surveillance of EOL issues. As a result of this process, we have a thorough understanding of current EOL surveys. In particular, we now know the topic areas for which items already exist and those topics that may require the development of new items. Therefore, our approach identified available items, and in some cases, the dearth of items, and is an efficient method to guide the process of developing surveillance items. For example, we could focus on pilot-testing existing items for their suitability for population-based surveillance and direct our limited resources to developing new items in topic areas that lack tested items.

We were able to locate 36 surveys containing EOL items. Ten of the 36 surveys were conducted in individual states, which indicates interest in this issue at the community level. Conversely, given that most deaths occur in hospitals and other health care settings, we were not surprised to find that most of the items (136 of 333) focused on different situational aspects of EOL care, such as where the respondent's loved one died and whether the dying person experienced pain at the end of his or her life. Similarly, more than half of the attitude-related items focused on the respondents' desires and expectations for EOL care, including their wishes for different aspects of EOL care.

We found few items that addressed respondents' knowledge and understanding of EOL options, such as hospice and palliative care. Furthermore, although we found many items related to the completion of advance directives and discussions about EOL preferences, few items asked whether respondents informed health care providers or family members that they had an advance directive. Studies (14,15) indicate that, even when people complete advance directives, these documents may not come to the attention of their health care providers. Surveillance data could be used to inform public health interventions that encourage communication between health care providers, patients, and family members about advance directives.

### Implications for public health practice

Surveillance of EOL issues may pose several challenges, some of which are not unique to this topic. For example, as with other sensitive health issues such as mental health or sexual behaviors, there are taboos associated with discussing EOL issues. Other data indicate that adults are more comfortable talking with their children about safe sex than discussing EOL issues with their parents (16). Recognizing these sensitivities when administering EOL items to respondents is necessary.

Another challenge is that EOL issues are usually considered within the context of the health care system. To a certain extent, this association is understandable, but as our prior work (9,11,17) illustrates, there are many ways for public health to contribute in this area. Because EOL issues are commonly associated with the health care system and are relatively new to the public health community, we have long recognized the importance of educating our public health colleagues about the potential roles that we can play in improving EOL experiences. As we move forward, educating our partners about the public health system and the range of activities that are part of public health practice is critical. For this project, we devoted time during our in-person meeting to educate the expert panel members about public health surveillance, including how data are used to inform public health activities and the costs associated with adding items to current surveillance systems. This approach has also been useful in working with partners to develop surveillance measures for other emerging public health concerns, such as cognitive health.

Finally, the current restrictions on the size of a typical surveillance module are another challenge. Current surveillance systems require that sets of questions focusing on a specific issue contain as few items as possible because of the administrative and implementation costs associated with surveillance procedures and to minimize burden on survey participants. Although we sought advice from experts regarding the critical topics for public health surveillance, these topics are not prioritized. A next step will be to use some type of metric to set priorities among the topics and the items within.

The limited size of a typical surveillance module may influence decisions whether to include retrospective or prospective EOL items. Many EOL surveys that focused on the quality of EOL care involved a retrospective or a mortality follow-back approach in which respondents are asked to provide information on the experiences of a family member or significant other who died within a specified time frame. We abstracted retrospective items that focused on various situational aspects of EOL care (eg, location of death). If we were to include these items on a surveillance module, another 1 or 2 screening items would be necessary to determine whether the respondent experienced the death of a loved one within a specific time frame and was familiar enough with the circumstances during this period to answer the question. Thus, a retrospective approach would have an impact on the total number of EOL items that could be included in the module as well as the number of respondents who could answer questions.

Conversely, a prospective approach may provide a view of EOL issues that complements previous surveys that have focused on this issue from a health care perspective. The entire sample could respond to prospective items, which may examine the respondents' knowledge, expectations, and behaviors with respect to EOL issues. Such data could help elucidate potential cultural differences regarding EOL planning and discussions and inform programs that target specific groups. Furthermore, periodic collection of population-based data on public attitudes and actions related to advance care planning would be useful in detecting potential changes that may occur when EOL issues are the focus of national attention (eg, 2008 National Health Care Decisions day, Terri Schiavo debate).

### Conclusions

We introduce a novel approach for identifying potential items for public health surveillance from the universe of existing questions on EOL issues. Using our environmental scan, we identified 333 items related to critical topics for public health surveillance of EOL issues. Information about the surveys and survey items has been placed in a database that summarizes the findings and provides information to others interested in EOL surveillance. In addition, we identified the gaps for which new items may be developed. We plan to ask state coordinators and policy makers for guidance in developing a smaller set of EOL items for cognitive and pilot testing and determining the need for developing new items. If EOL items are included in population-based surveillance systems, they have the potential to yield information that will provide a broader perspective of EOL issues than has been available to date.

## Figures and Tables

**Table 1:** Characteristics of Surveys^a^ (N = 36), End-of-Life Survey Scan, 2006

Characteristic	Value
**Setting (n)**	
National	8
State	10
Community	4
Hospital	9
Hospice	4
Nursing Home	1
**Sample (n)**	
Public	20
Patient group	5
Family members	11
**Mode of administration (n)**	
In person	14
Telephone	13
Written	8
Telephone and written	1
Total no. of end-of-life survey items	1495^b^
Items abstracted (n)	333
**Response type (no. of survey items)**	
Yes/no	133
Multiple choice	111
Rating	37
Likert scale	42
Open-ended	10
**Perspective (no. of items)**	
Prospective	159
Retrospective	174
**Overarching categories (no. of items)**	
Knowledge	16
Attitudes	124
Behavior	57
Situation	136

a Surveys retrieved through searches of the Internet and published and "gray" (articles, technical reports, newsletters, or other documents produced by governmental agencies, academic institutions, and other groups not indexed or distributed by commercial publishers) literature, as well as from experts. The list of 36 surveys is available on request from the authors.

b Range was 4 to 126 items per survey.

**Table 2:** Distribution of 333 End-of-Life (EOL) Items Within Priority Topics for Public Health Surveillance, End-of-Life Survey Scan, 2006

Overarching Category With Subtopic	Definition of Subtopic	Sample Item	Perspective	No. of Items
**Knowledge^a^ (n = 16)**				
Awareness	Knowledge or understanding of EOL options	Do you believe you need more information in order to make the best decisions for your EOL care?	Prospective	16
**Attitude^b^ (n = 124)**				
Burden	Concerns about being a burden to others	I am concerned about becoming a burden physically or emotionally on my family because of my illness.	Prospective	11
Concerns and Fears	General concerns and fears about death or dying	I am concerned that my life will be inappropriately prolonged by the use of machines.	Prospective	35
		How much, if at all, does each of these medical matters worry you when you think about your death? The possibility of great physical pain before you die	Retrospective	3
Desires	Expectations about EOL care	How important would each of the following be to you when dealing with your own dying? [Choosing your treatment options]	Prospective	65
		Did [the patient] have specific wishes or plans about the types of medical treatment (he/she) wanted while dying?	Retrospective	10
**Behavior^c^ (n = 57)**				
Communications	Communication with health care provider or family members about advance directive	Who, if anyone, have you told that you have signed either or both of these documents?	Prospective	2
		Had you or [the patient] discussed a living will or durable power of attorney for health care with a doctor caring for [the patient]?	Retrospective	2
Completed an advance directive	Completion of advance directives, living will, or durable power of attorney	Can you tell us why you do not have a living will?	Prospective	18
		Do you have written instructions about the type of medical treatment you would want to receive if you were unconscious or somehow unable to communicate?	Retrospective	18
Discuss EOL	Discussion of end-of-life preferences and options with others	Have you ever discussed with your doctor how you would want to be treated if you were dying?	Prospective	12
		Did you or [the patient] and the hospice team make a plan to ensure that any wishes [the patient] had for medical care were followed?	Retrospective	5
**Situation^d^ (n = 136)**				
Location	Location of death of loved one	During the last 3 months this person was alive, did he/she receive care through a hospice?	Retrospective	24
Needs	How well was this person's needs met	Were any of the prescribed pain medications that this person was supposed to use difficult to obtain at a local pharmacy?	Retrospective	60
Pain	Experience of pain at the end of patient's life	During [the patient's] last month of life, how much of the time did [the patient] experience pain?	Retrospective	25
Provider	Health care provider communication with dying person or family members	How often were you and [the patient] able to talk to doctors and others who took care of [the patient] when you needed to?	Retrospective	27

a Overarching category that involved questions about respondent's understanding.

b Overarching category that involved questions about respondent's beliefs.

c Overarching category that involved questions about respondent's actions.

d Overarching category that involved questions about respondent's perceptions during death of loved one.
